# Novel familial distal imprinting centre 1 (11p15.5) deletion provides further insights in imprinting regulation

**DOI:** 10.1186/s13148-019-0629-x

**Published:** 2019-02-15

**Authors:** Florian Kraft, Katharina Wesseler, Matthias Begemann, Ingo Kurth, Miriam Elbracht, Thomas Eggermann

**Affiliations:** Institute of Human Genetics, Medical Faculty, RWTH Aachen University, Pauwelsstr. 30, D-52074 Aachen, Germany

**Keywords:** Beckwith-Wiedemann syndrome, Silver-Russell syndrome, Imprinting centre 1, H19/IGF2:IG-DMR, Deletion, Nanopore sequencing

## Abstract

**Background:**

Deletions of the imprinting centre 1 (IC1) in 11p15.5 are rare and their clinical significance is not only influenced by their parental origin but also by their exact genomic localization. In case the maternal IC1 allele is affected, the deletion is associated with the overgrowth disorder Beckwith-Wiedemann syndrome (BWS) and a gain of methylation (GOM) of the IC1. The consequences of deletions of the paternal IC1 allele depend on the localization and probably the binding sites of methylation-specific DNA-binding factors affected by the change. It has been suggested that distal deletions of the paternal allele are associated with a normal IC1 methylation and phenotype, whereas proximal alterations cause a loss of methylation (LOM) and Silver-Russell syndrome (SRS) features.

**Results:**

In a patient referred for molecular BWS testing and his family, a deletion within the IC1 was identified by MLPA. It was associated with a GOM, corresponding to the transmission of the alteration via the maternal germline. Accordingly, the deletion was also detectable in the maternal grandmother, but here the paternal chromosome 11p15.5 was affected and a IC1 LOM was observed. By nanopore sequencing, the localization of the deletion could be precisely determined.

**Conclusions:**

We report for the first time both GOM and LOM of the IC1 in the same family, caused by transmission of a 2.2-kb deletion in 11p15.5. Nanopore sequencing allowed the precise characterization of the change by long-read sequencing and thereby provides further insights in the regulation of imprinting in the IC1.

**Electronic supplementary material:**

The online version of this article (10.1186/s13148-019-0629-x) contains supplementary material, which is available to authorized users.

## Background

The chromosomal region 11p15.5 harbours two imprinting control regions (ICs), the telomeric IC1 (with the differentially methylated region (DMR) H19/IGF2:IG-DMR) and the centromeric IC2 (including the KCNQ1OT1:TSS-DMR).

Molecular alterations of both regions are associated with two imprinting disorders, the overgrowth disease Beckwith-Wiedemann syndrome (BWS, OMIM130650) and the growth retardation disorder Silver-Russell syndrome (SRS, OMIM180860). On a molecular level, the contrary growth features of BWS and SRS are reflected by opposing mutations and epimutations (for review: [1]). Duplications of the maternal allele at chromosome 11p15.5 result in SRS whereas duplications of the paternal allele are associated with BWS. Furthermore, BWS patients can exhibit a gain of methylation (GOM) of the IC1 whereas the loss of methylation (LOM) of the same IC leads to SRS.

Deletions restricted to the IC1 are rare and their clinical significance is not only influenced by their parental origin but also by the exact genomic localization (for review: [2]). In fact, overgrowth phenotypes and BWS features are linked to deletions of the maternal IC1 allele resulting in GOM of the H19/IGF2:IG-DMR which itself is not affected. Paternal inheritance of the deletion in these families is associated with normal methylation and does not exhibit apparent pathologic phenotypes (for review: [2]). However, Abi Habib et al. [3] described three SRS patients with deletions affecting the paternal IC1 allele. To explain this contradictory observation of both SRS and normal development in the case of paternal transmission of IC1 deletions, Sparago et al. [2] recently suggested that the clinical phenotype does depend not only on the parental origin of the affected allele, but also on the transcription factor binding sites disturbed by the deletion.

The IC1 regulates the expression of the paternally expressed growth factor IGF2 and the maternally expressed *H19* gene by differential methylation of the H19/IGF2:IG-DMR. It includes binding sites for methylation-specific DNA binding factors (for review: [2]). In the unmethylated state, the maternal IC1 enables the binding of the CCCTC-binding factor (CTCF). This binding is required to maintain the unmethylated status of the allele and to inhibit the interaction of the *IGF2* promotors with enhancer motifs that are shared between *IGF2* and *H19* (for review: [4]). Conversely, the paternal IC1 copy is methylated, thereby preventing the binding of CTCF and allowing the expression of *IGF2*. The maintenance of the paternal IC1 methylation marks is mediated by the KRAB zinc finger protein ZFP57. Thus, there is a cluster of CTCF/ZFP57 binding sites (BS) in the repetitive regions of the IC1. Germline mutations in *ZFP57* have previously been identified to cause LOM of specific genes and are associated with the imprinting disorder transient neonatal diabetes mellitus [5], but so far have not linked to SRS or BWS [6, 7]. Furthermore, the IC1 harbours binding sites for additional factors mediating the proper methylation status of the region, i.e. YY1, OCT4/SOX2 and ZBTB33 (KAISO) [8–10].

We report for the first time both gain and loss of methylation of the H19/IGF2:IG-DMR in the same family, caused by transmission of a 2.2-kb deletion within the IC1. Nanopore sequencing allowed the precise characterization of the aberration by long-read analysis, thereby providing further insights in the regulation of imprinting in the IC1.

### Patient and family

**Fig. 1:**
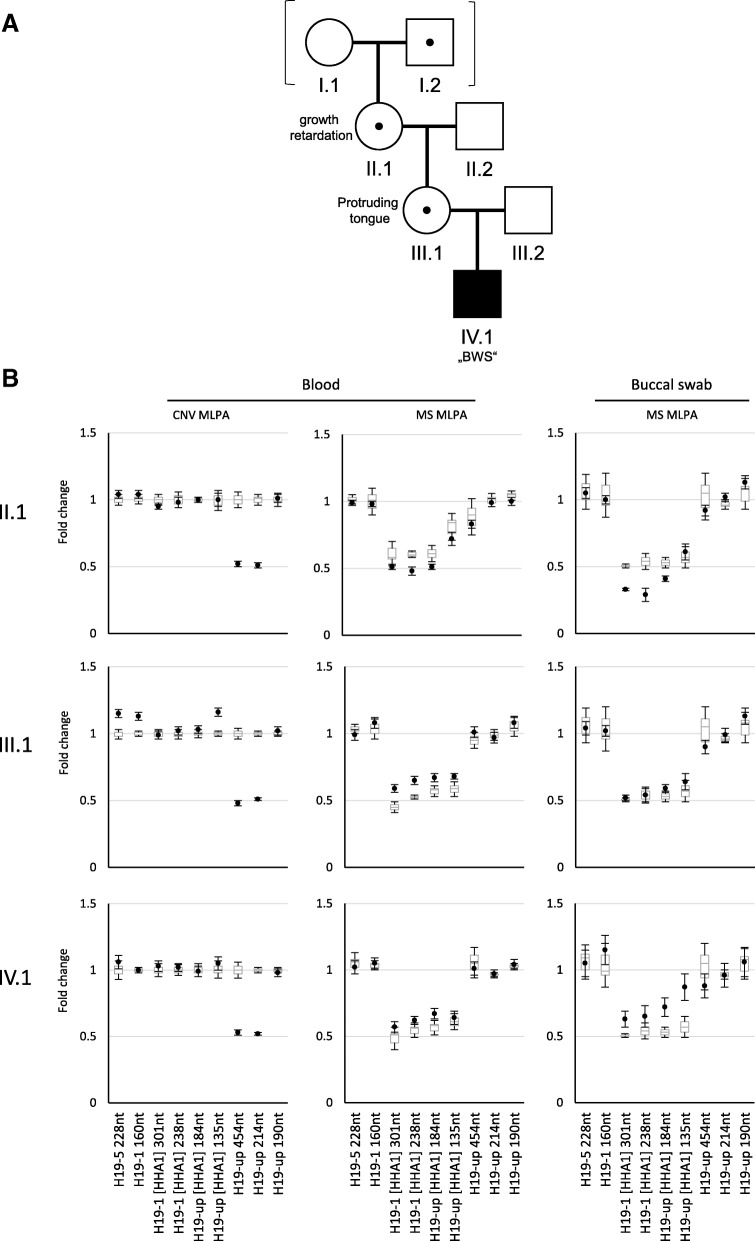
MS MLPA results showing GOM and LOM of the H19/IGF2:IG-DMR within the same family (**a**) with a deletion within the IC1. (**b**) In the MLPA copy number run (CNV MLPA), heterozygosity for the deletion is visible, whereas in the methylation specific run (MS MLPA) either LOM or GOM could be demonstrated. It should be noted that the MS MLPA probes are not affected by the deletion. The findings from lymphocyte analysis could be confirmed in buccal swab DNA. (Box plots showing the first to the third quartile of the data from healthy controls. The horizontal line with in the plots marks the median copy number or methylation, respectively. The data from the patients are shown as black dots. The whiskers of the box plots and dots indicate the standard deviation)

The patient (Fig. 1a (IV.1)) was referred for molecular testing with the clinical diagnosis of BWS. He was born to unrelated German parents after an uneventful pregnancy at 39 + 6 gestational week. Birth was complicated by arrest of labour, and the newborn showed temporary hemiplegia of left body half. Birth length was 52 cm (*z* − 0.13), birth weight 3615 g (z 0.04) and head circumference at birth was (OFC) 33 cm (z − 1.07). Mild body asymmetry was documented with a slightly longer right leg. The boy showed an open mouth with protruding tongue and macroglossia.

At time of molecular diagnostics (age 3 1/12 years), body measurements were within the upper normal range. Length was 102 cm (*z* 1.22), weight 17 kg (*z* 1.14) and OFC 50 cm (*z* − 0.56).

Other features characteristic for BWS like exomphalos, hyperinsulinism or tumours were not reported. However, based on the recently published BWS score, the presence of even slight (lateralized) overgrowth and/or macroglossia as cardinal features merits molecular testing for BWS [11].

The mother of the index patient (Fig. 1a (III.1)) was born at term with birth measurements within the normal range (birth length 54 cm (*z* 1.05), birth weight 3400 g (*z* − 0.17), and a protruding tongue. Her growth was unremarkable with a final height of 166 cm (z 0.41). She did not exhibit obvious cardinal features of BWS, but a third kidney was reported. Her father had a final height of 183 cm.

Growth parameters of the patients’ maternal grandmother (Fig. 1a (II.1)) were reported in the lower normal range in infancy and childhood, and her final height was 156 cm. Her weight was markedly reduced at 7 years (12 kg, *z* − 5.84). On photographs from early childhood body, a protruding forehead was apparent. Unfortunately, further data were not available due to the poor documentation of clinical data at time of birth of the proband, and therefore the clinical Netchine-Harbison score for SRS could not be applied [12].

## Results

In a patient referred for molecular BWS testing, a deletion within the H19/IGF2:IG-DMR was observed in peripheral lymphocytes by multiplex ligation probe-dependent amplification (MLPA) in two independent runs, affecting the H19 copy number probes 10588-L11143 and 10586-L11141 and spanning at least 500 bp (GRCh37/hg19:chr11:2,022,347–2,022,846) (Fig. 1). The four methylation-specific (MS) H19 probes (GRCh37/hg19:2,019,408–2,019,720) in the MLPA showed a normal copy number but revealed a borderline GOM of the H19/IGF:IG-DMR, indicating that the maternal allele was affected by the deletion. This assumption was confirmed by identification of the same MLPA pattern in the maternal DNA sample, also associated with a GOM.

In the maternal grandmother, the deletion could be detected as well, but here MS MLPA revealed that the paternal chromosome 11p15.5 was affected. Similar patterns could be observed in DNA from buccal swabs of the family.

The deletion in the family could be confirmed by SNP array analysis, revealing heterozygosity for a IC1 deletion with a size of 3 kb in maximum (arr[hg19] 11p15.5(2021892-2024683)× 1).

**Fig. 2:**
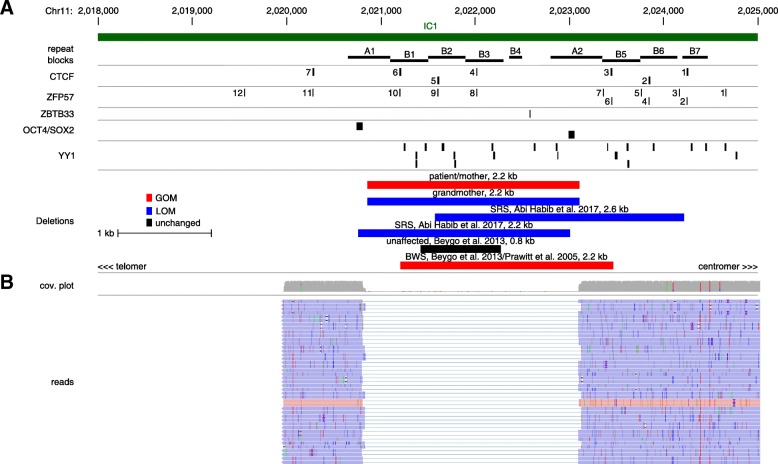
The exact position of the pathogenic IC1 deletion could be identified by nanopore sequencing and alters transcription factor binding sites (https://genome.ucsc.edu/). **a** UCSC custom track of the IC1 in 11p15.5 (hg19), illustrating the localization of the A and B type repeats as well as CTCF, ZFP57, ZBTB33, OCT4/SOX2 and YY1 binding sites (different distributions on forward and reverse strand are not shown). Additionally, the localization of the deletion in our family and in selected patients with IC1 deletions from the literature are shown (black boxes: position of transcription factor binding sites and repeats; red horizontal bars: deletions leading to GOM; blue horizontal bars: deletions associated with LOM; black horizontal bars: unchanged methylation). **b** IGV view from the affected region of the IC1 showing the coverage plot and some of the reads from the nanopore sequencing harbouring the deletion

By long-range PCR, two PCR products could be generated, one with a size of ~ 6 kb which occurred in the control as well as in the patients’ DNA samples, and a ~ 4 kb detectable only in the deletion carrier. By nanopore sequencing, the size of the deletion could be determined as 2209 bp, and affecting the region 2,020,859–2,023,111 on chromosome 11 (GRCh37/hg19) (Fig. 2). The region harbours the CTCF BSs 4 to 6, the ZFP57 BSs 8 to 10 and 12 potential YY1 BS (Fig. 2). The breakpoints are located within the centromeric A1 and the telomeric A2 repeat, and therefore, the deletion comprises the repeat elements B1 to B4.

## Discussion

Regulation of the IC1 in 11p15.5 requires a precise arrangement of binding sites for different transcription factors as the prerequisite for a proper imprint mark and expression of imprinted genes [2, 13]. Valuable insights in this complex regulation are provided by patients with copy number variants within the IC1 and their families. In fact, duplications affecting either the maternal or the paternal H19/IGF2:IG-DMR alleles have already been reported, and clinically, these patients exhibit opposite features [14, 15]. However, the situation is different in the case of deletions within the IC1: In families with both paternal and maternal inheritance, only IC1 GOM associated with BWS-like occurred in case the maternal allele was affected, whereas a paternal transmission was associated with a normal methylation and phenotype (for review: [2]). In contrast, deletions localized more proximally have been suggested to cause an IC1 LOM and SRS features in case the paternal allele is affected [3], but up to now, the consequences of these deletions in the case of maternal transmission could not be demonstrated.

Here we report on the first family with both IC1 GOM and LOM in three generations, in which the altered imprints depend on the sex of the parent contributing the IC1 deletion. Precise mapping of the alteration indicates that it is the most distal deletion associated with IC1 GOM described so far, and it exhibits similar breakpoints as a recently published SRS patient with IC1 LOM [3]. The resulting arrangement of transcription factor binding sites may explain the occurrence of both epigenotypes in the same family, as well as the associated clinical features [2]. In contrast to the obvious effect of the deletion on the methylation status of the H19/IGF2:IG-DMR, the clinical features of the deletion carriers are only subtle. Furthermore, the index patient (Fig. 1 (IV.1)) exhibited a body and limb asymmetry. Both observations are at first glance difficult to explain because the 11p15.5 deletion is transmitted in the family and mosaicism which might explain the clinical heterogeneity and the asymmetry can be excluded. Similar clinical observations have been reported the SRS family with a 2.2-kb deletion affecting the same genomic region [3] as well as in BWS families [13] and might be caused by epigenetic mosaicism on individual cell level as suggested by [3].

The influence of the sex of the parent contributing to the affected allele as well as the size and localization of the deletion on the H19/IGF2:IG-DMR methylation and the phenotype was systematically analysed by Beygo et al. [13]. Based on DNA methylation analyses of the IC1 and CTCF binding studies, Beygo and colleagues [13] hypothesized that H19/IGF2:IG-DMR methylation depends on the spatial arrangement of the remaining CTCF BSs. It was postulated that deletions resulting in CTCF BSs clusters longer than the wildtype clusters are associated with a GOM of the H19/IGF2:IG-DMR and a highly penetrant BWS phenotype, whereas microdeletions not altering the length of the CTCF BS cluster should have a milder consequence [13]. The observations in our family are consistent with this suggestion: the total distal CTCF BS cluster is lost (Fig. 2), but in the proximal CTCF BS cluster, only the A2 repeat is altered. Accordingly, only a slight GOM and single BWS features are present in the patient and his mother (Fig. 1 (IV.1, III.1)).

In addition, the deletion also affects ZFP57 binding which is responsible for the maintenance of the paternal H19/IGF2:IG-DMR methylation. Accordingly, LOM was detectable in the patients’ grandmother (Fig. 1 (II.1)). The deletion is thus comparable with a recently published SRS patient [3] by Sparago et al. [2]. They postulate that deletions strongly affecting ZFP57 binding regions result in LOM and SRS whereas deletions comprising less ZFP57 BSs do not alter methylation and phenotype.

The LOM of IC1 in the patients’ grandmother (II.1) and patients from the literature might also be caused by the loss of binding sites for YY1, ZBTB33 or OCT4/SOX2 (Fig. 2). In fact, YY1 has been suggested to be involved in maintaining the proper methylation status of differentially methylated regions [10]. In mice, YY1 has been shown to control the *Peg3* and *Gnas* imprinting domains, but it is also conceivable that it plays a broader role in the regulation of other imprinted regions. Binding of ZBTB33 (KAISO) is also regarded as relevant for methylation maintenance [9], whereas OCT4/SOX2 binding has been shown to prevent a gain of methylation [8].

Based on the identification of the first family with both IC1 LOM and GOM due to the switch of parental transmission of a microdeletion allele within 11p15.5, we confirm the hypothesis of Sparago and colleagues [2] that the specific parts of the IC1 harbour binding sites for different factors maintaining both the maternal or paternal imprint. The loss of these motifs results either in GOM or LOM, but the severity of the disturbed methylation and the penetrance of the phenotype depend on the spatial arrangement of the remaining binding sites in the IC1 [13]. In addition to the insights in the IC1 regulation, this report also illustrates the necessity of a careful characterization of IC1 microdeletions as the basis for a precise prediction of recurrence risks and clinical phenotypes.

## Material and methods

Blood samples were available from the patient, his parents and maternal grandfather. In addition, DNA from buccal swabs could be obtained from the patient, his mother and maternal grandmother. DNA was isolated according to standard protocols.

As first routine screen to detect copy number variations (CNVs) and aberrant methylation patterns in the 11p15.5 region, MS MLPA was performed according to the manufacturer’s instruction (kit ME030-C3, mrc Holland, Amsterdam/NL) (Additional files 1, 2 and 3). To confirm the deletion, a SNP array analysis (CytoScan® HD Array, Affymetrix, Santa Clara, CA, USA) was carried out.

To further determine the size of the deletion and for precise genomic mapping, a junction fragment was generated by long-range PCR (LR-PCR) covering the H19/IGF2:IG-DMR (forward primer: 5′-CTCTGGGATGTGGAAGGGC-3′; reverse primer: 5′-AATAGCCCGAGGTGTTTGCC-3′). LR-PCR was carried out in a 25-μl reaction containing 2.5 μl 10× Buffer II, 1 μl of fw and rev primer, 0.25 μl AccuPrime Taq (ThermoFisher, #12339016) and 40 ng of DNA. Amplification cycles consisted of 94 °C 02:00 min, (94 °C 00:30 min, 60 °C 00:30 min, 68 °C 06:00 min) × 34, 72 °C 10:00 min. Due to the repetitive nature of the region, we applied nanopore sequencing for analysing the PCR products as this allows sequencing of the complete PCR product at once. PCR products were prepared from agarose gels and used for nanopore library preparation kit LSK-108 (Oxford Nanopore Technologies, Oxford/UK). The library was sequenced on a GridION sequencer using a R9.4.1 flow cell (Oxford Nanopore Technologies). Data were processed, analyzed and visualized by nanopore tool box components (guppy, porechop, minimap2, canu, nanopolish) and the IGV browser (http://software.broadinstitute.org/software/igv/).

## Additional files

Additional file 1: **Figure S1.** Results of the CNV and MS MLPA of the index patient IV.1 (A) CNV and (B) MS MLPA from the blood. (C) MS MLPA from the buccal swab. (box plots showing the first to the third quartile of the data from healthy controls. The horizontal line with in the plots marks the median. The data from the patients are shown as black dots. The whiskers of the box plots and dots indicate the SD). (PDF 203 kb)

Additional file 2: **Figure S2.** Results of the CNV and MS MLPA of the mother III.1. (A) CNV and (B) MS MLPA from the blood. (C) MS MLPA from the buccal swab. (box plots showing the first to the third quartile of the data from healthy controls. The horizontal line with in the plots marks the median. The data from the patients are shown as black dots. The whiskers of the box plots and dots indicate the SD). (PDF 203 kb)

Additional file 3: **Figure S3.** Results of the CNV and MS MLPA of the grandmother II.1. (A) CNV and (B) MS MLPA from the blood. (C) MS MLPA from the buccal swab. (box plots showing the first to the third quartile of the data from healthy controls. The horizontal line with in the plots marks the median. The data from the patients are shown as black dots. The whiskers of the box plots and dots indicate the SD). (PDF 203 kb)

## Abbreviations

BS: Binding site
BWS: Beckwith-Wiedemann syndrome
DMR: Differentially methylated region
GOM: Gain of methylation
IC: Imprinting centre
IC1: Imprinting centre 1 (in 11p15.5)
IC2: Imprinting centre 2 (in 11p15.5)
LOM: Loss of methylation
MLPA: Multiplex ligation-dependent probe amplification
SRS: Silver-Russell syndrome
