# Supplementary figures and images for: Novel familial distal imprinting centre 1 (11p15.5) deletion provides further insights in imprinting regulation
Source: Clin Epigenetics. 2019 Feb 15;11:30. doi: 10.1186/s13148-019-0629-x (PMC6377752; doi:10.1186/s13148-019-0629-x)

Suppl. Fig. 1

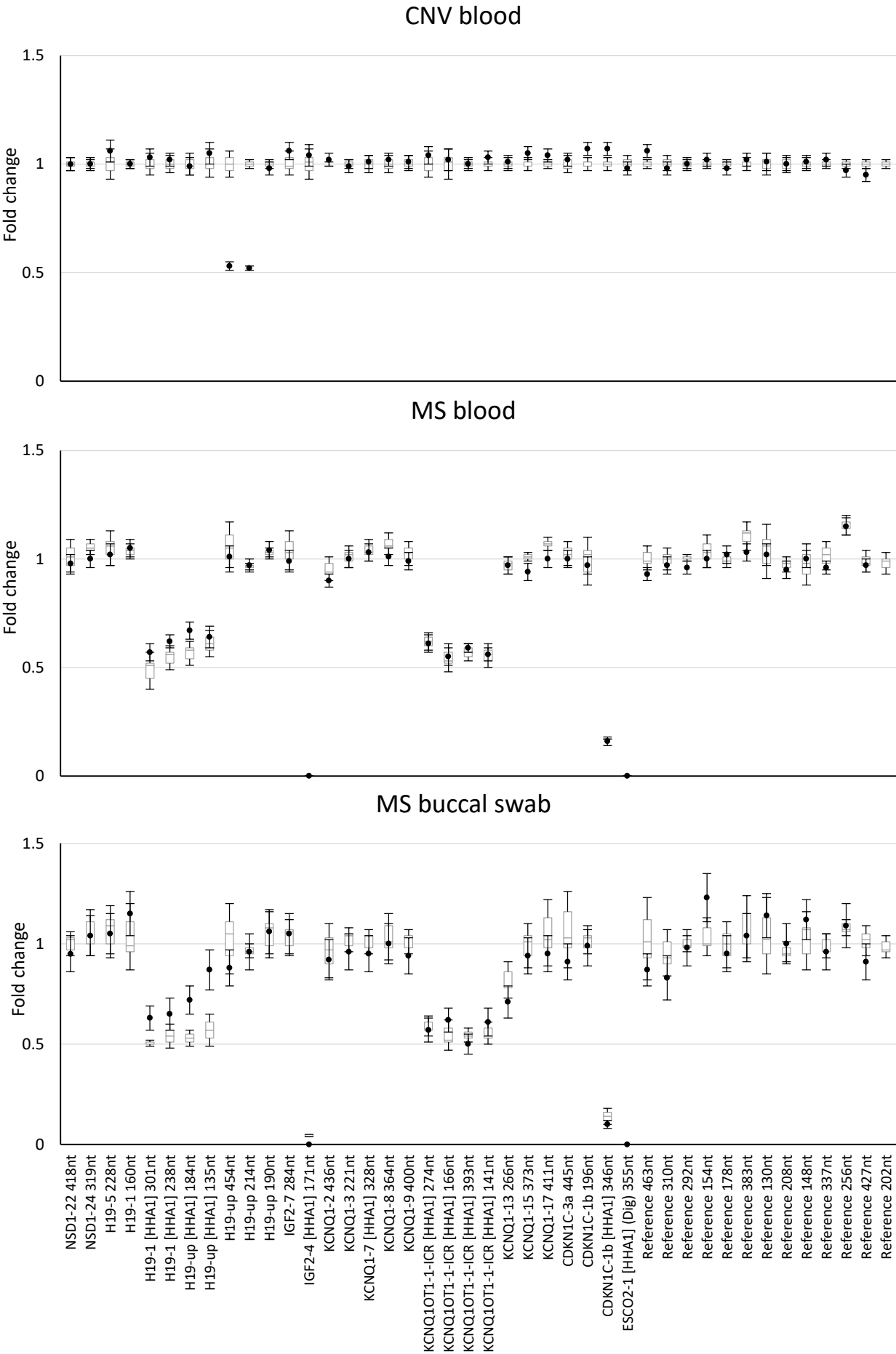

Supplement: Supplementary file 1 — Figure S1. Results of the CNV and MS MLPA of the index patient IV.1 (A) CNV and (B) MS MLPA from the blood. (C) MS MLPA from the buccal swab. (box plots showing the first to the third quartile of the data from healthy controls. The horizontal line with in the plots marks the median. The data from the patients are shown as black dots. The whiskers of the box plots and dots indicate the SD). (PDF 203 kb) [file 13148_2019_629_MOESM1_ESM.pdf]

Suppl. Fig. 2

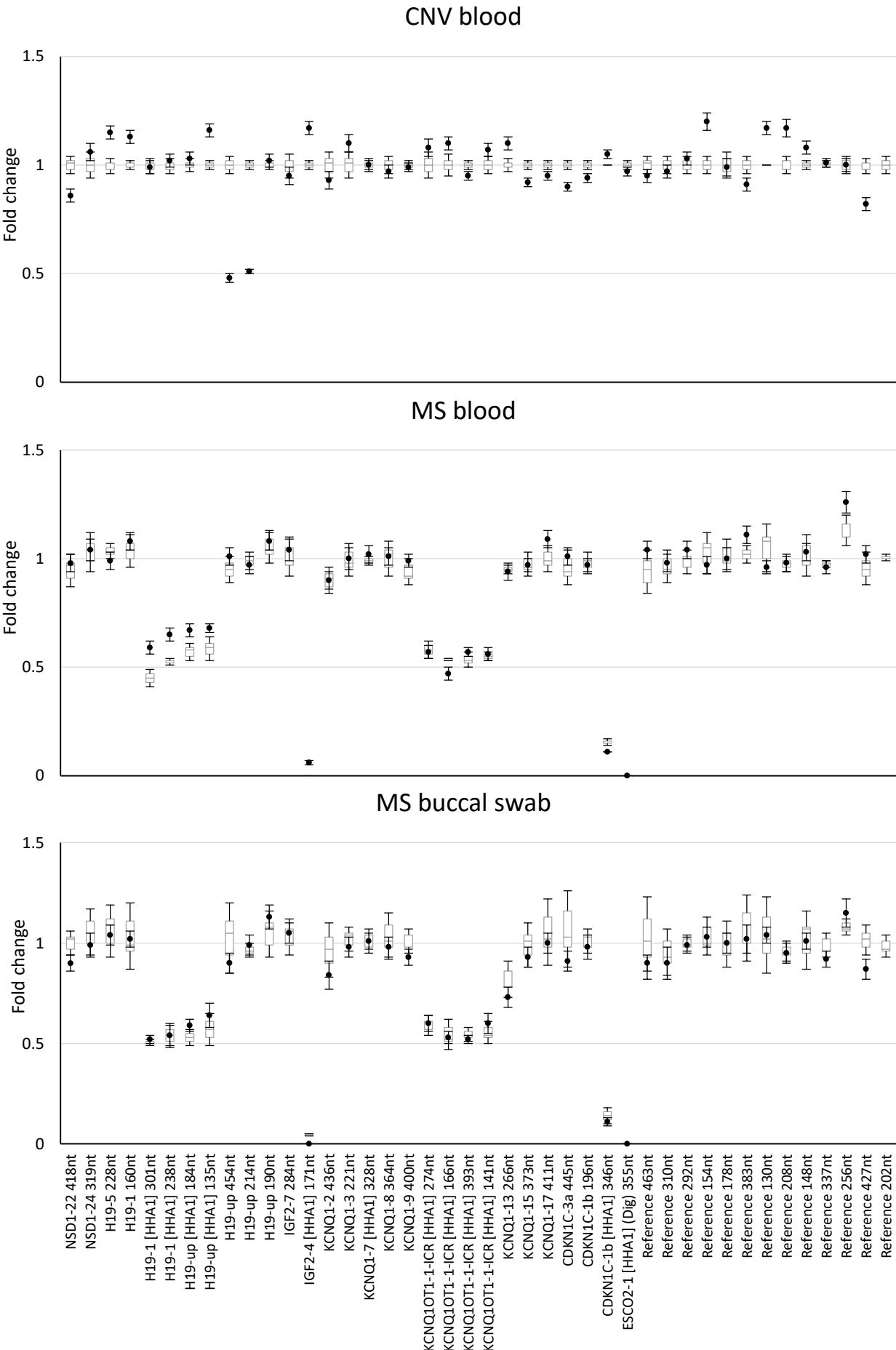

Supplement: Supplementary file 2 — Figure S2. Results of the CNV and MS MLPA of the mother III.1. (A) CNV and (B) MS MLPA from the blood. (C) MS MLPA from the buccal swab. (box plots showing the first to the third quartile of the data from healthy controls. The horizontal line with in the plots marks the median. The data from the patients are shown as black dots. The whiskers of the box plots and dots indicate the SD). (PDF 203 kb) [file 13148_2019_629_MOESM2_ESM.pdf]

Suppl. Fig. 3

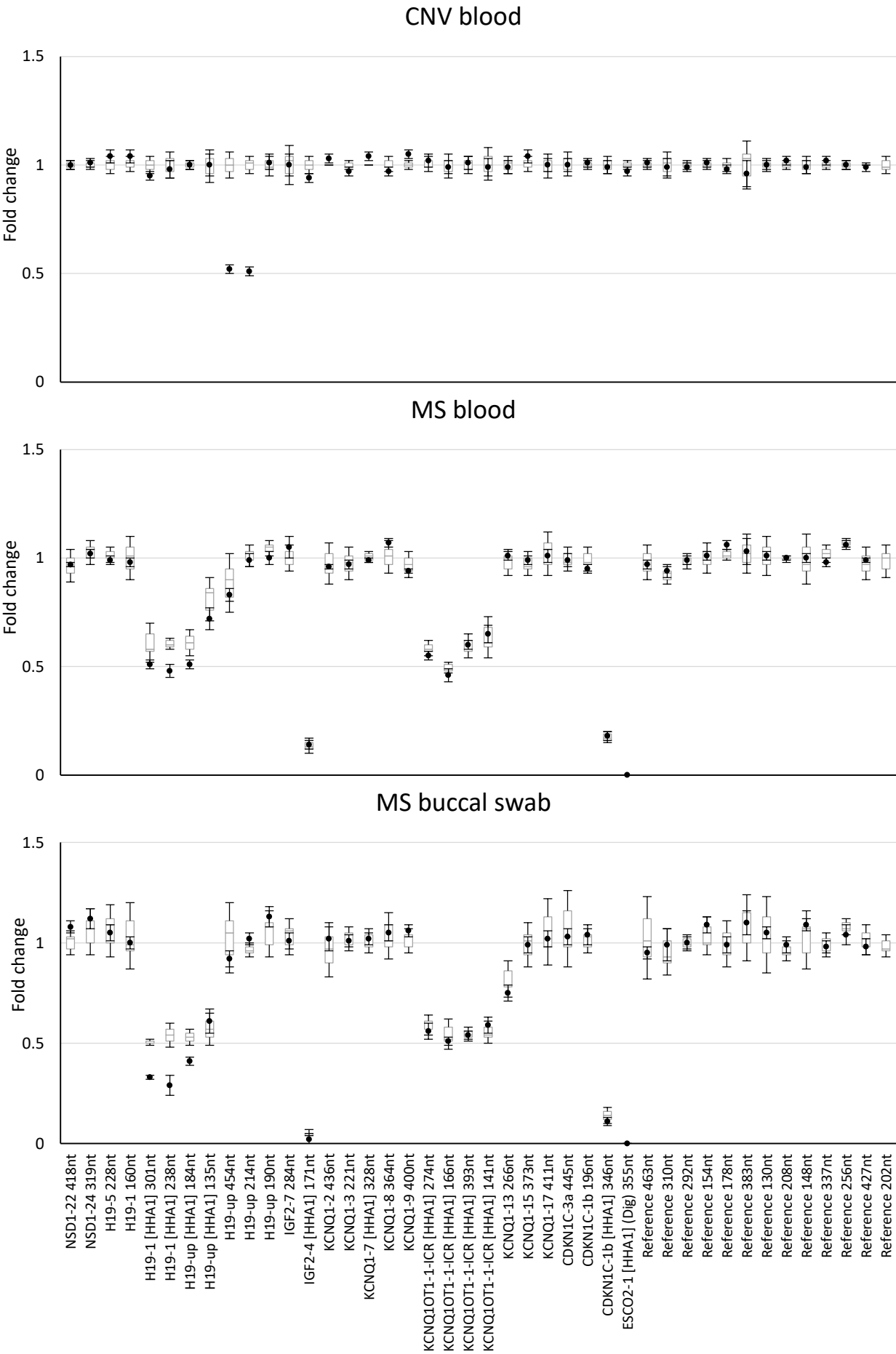

Supplement: Supplementary file 3 — Figure S3. Results of the CNV and MS MLPA of the grandmother II.1. (A) CNV and (B) MS MLPA from the blood. (C) MS MLPA from the buccal swab. (box plots showing the first to the third quartile of the data from healthy controls. The horizontal line with in the plots marks the median. The data from the patients are shown as black dots. The whiskers of the box plots and dots indicate the SD). (PDF 203 kb) [file 13148_2019_629_MOESM3_ESM.pdf]
